# Metformin Suppresses the Progress of Diabetes-Accelerated Atherosclerosis by Inhibition of Vascular Smooth Muscle Cell Migration Through AMPK–Pdlim5 Pathway

**DOI:** 10.3389/fcvm.2021.690627

**Published:** 2021-07-23

**Authors:** Yi Yan, Ting Li, Zhonghao Li, Mingyuan He, Dejiang Wang, Yingyi Xu, Xuewen Yang, Yuanyuan Bai, Yi Lao, Zhiyong Zhang, Wei Wu

**Affiliations:** ^1^Department of Cardiology, Translational Research Centre of Regenerative Medicine and 3D Printing of Guangzhou Medical University, The Third Affiliated Hospital of Guangzhou Medical University, Guangzhou, China; ^2^State Key Laboratory of Organ Failure Research, Southern Medical University, Guangzhou, China; ^3^Department of Pathophysiology, Key Lab for Shock and Microcirculation Research of Guangdong, Southern Medical University, Guangzhou, China; ^4^Guangzhou First People's Hospital, Guangzhou, China; ^5^Department of Cardiology, Zhongshan Hospital of Sun Yat-sen University, Zhongshan, China

**Keywords:** metformin, Pdlim5, AMPK, diabetes, vascular smooth cells

## Abstract

**Backgrounds:** Our previous work revealed that AMP-activated protein kinase (AMPK) activation inhibits vascular smooth muscle cell migration *in vitro* by phosphorylating PDZ and LIM domain 5 (Pdlim5). As metformin is an AMPK activator, we used a mouse vascular smooth muscle cell (VSMC) line and a *Myh11-cre-EGFP* mice to investigate whether metformin could inhibit the migration of VSMCs *in vitro* and in a wire-injury model *in vivo*. It is recognized that VSMCs contribute to the major composition of atherosclerotic plaques. In order to investigate whether the AMPK–Pdlim5 pathway is involved in the protective function of metformin against atherosclerosis, we utilized ApoE^−/−^ male mice to investigate whether metformin could suppress diabetes-accelerated atherosclerosis by inhibition of VSMC migration *via* the AMPK–Pdlim5 pathway.

**Methods:** The mouse VSMC cell line was exogenously transfected wild type, phosphomimetic, or unphosphorylatable Pdlim5 mutant before metformin exposure. *Myh11-cre-EGFP* mice were treated with saline solution or metformin after these were subjected to wire injury in the carotid artery to study whether metformin could inhibit the migration of medial VSMCs into the neo-intima. In order to investigate whether the AMPK–Pdlim5 pathway is involved in the protective function of metformin against atherosclerosis, ApoE^−/−^ male mice were divided randomly into control, streptozocin (STZ), and high-fat diet (HFD)-induced diabetes mellitus; STZ+HFD together with metformin or Pdlim5 mutant carried the adenovirus treatment groups.

**Results:** It was found that metformin could induce the phosphorylation of Pdlim5 and inhibit cell migration as a result. The exogenous expression of phosphomimetic S177D-Pdlim5 inhibits lamellipodia formation and migration in VSMCs. It was also demonstrated that VSMCs contribute to the major composition of injury-induced neointimal lesions, while metformin could alleviate the occlusion of the carotid artery. The data of ApoE^−/−^ mice showed that increased plasma lipids and aggravated vascular smooth muscle cell infiltration into the atherosclerotic lesion in diabetic mice were observed Metformin alleviated diabetes-induced metabolic disorders and atherosclerosis and also reduced VSMC infiltration in atherosclerotic plaques, while the Pdlim5 phospho-abolished mutant that carried adenovirus S177A-Pdlim5 undermines the protective function of metformin.

**Conclusions:** The activation of the AMPK–Pdlim5 pathway by metformin could interrupt the migratory machine of VSMCs and inhibit cell migration *in vitro* and *in vivo*. The maintenance of AMPK activity by metformin is beneficial for suppressing diabetes-accelerated atherosclerosis.

## Introduction

Atherosclerosis is a chronic artery disease and responsible for one in four deaths induced by cardiovascular diseases (1, 2). Atherosclerosis is initiated with a regional endothelial injury and followed by monocyte adhesion, infiltration, and differentiation into macrophages, with the latter one taking up oxidized LDL and becoming foam cell (3–6). However, the major cell type in atherosclerotic plaques is vascular smooth muscle cell (VSMC), which can also become a foam cell (7). The VSMCs accumulated in the intima are thought to be the major source of extracellular matrix (ECM) and foam cells in fatty streaks (pre-atherosclerotic plaques) (3, 8). With time, these early fatty streak lesions develop into advanced lesions, some of which will eventually become unstable and rupture, resulting in the adverse clinical events of cardiovascular disease (CVD) (7, 9, 10). As described above, atherosclerotic lesions are formed through the complex interactions of various factors, and insulin resistance and hyperglycemia in diabetes mellitus (DM) accelerate all these interactions, with greater vascular inflammation, larger necrotic core, and more diffuse atherosclerosis in the coronary arteries (3). The underlying mechanism is still not very clear, probably due to the excessive and prolonged production of reactive oxidative species (10). Many clinical trials showed that intensive glucose therapy in patients with type 2 diabetes mellitus (T2DM) reduces the risk of a cardiovascular disease (11, 12). Even in individuals with prediabetes, the risk of CVD was increased (13). It was found that metformin, a hypoglycemic agent, exhibits abilities to suppress the progression of common carotid intima-media thickness in T2DM patients and also reduces the incidence of myocardial infarction (11, 12, 14, 15). However, it is paradoxical that several recent clinical trials showed that the anti-atherogenic effect of metformin seems independent of its hypoglycemic function because other regular therapies, such as insulin and sulfonylurea, possess less beneficial cardiovascular effects (16–19). A possible target of metformin is AMP-activated protein kinase (AMPK), a cellular energy sensor activated under metabolic stress (20). It has been reported that the activation of AMPK by metformin reduces endothelial mitochondrial fragmentation and suppresses atherosclerotic plaques in diabetic mice (15). Our previous findings found that AMPK phosphorylates PDZ and LIM domain 5 (Pdlim5), a protein involved in cytoskeleton organization, on Ser177 to inhibit vascular smooth muscle cell migration by suppressing the Rac1-Arp2/3 signaling pathway (21). Recent genetic lineage tracing studies showed that VSMCs get involved in every stage of atherosclerosis development and are a major cell type in an atherosclerotic plaque (8). The single-cell omics also reveals the heterogeneity and plasticity of VSMCs in the vessel wall during atherogenesis (22). The VSMCs not only take part in ECM synthesis and fibrous cap formation but also switch into macrophage-like cells/foam cells and even contribute to calcification and necrotic core, which are crucial to plaque instability (8, 23, 24). Considering the complexity of the roles that VSMCs played in the development of atherosclerosis and the importance of VSMCs in atherosclerotic plaques, we assume that activation of the AMPK–Pdlim5 pathway by metformin may be beneficial for suppressing diabetes-accelerated atherosclerosis *via* the inhibition of VSMC migration. In this study, we identified that metformin could induce the phosphorylation of Pdlim5 at Ser177 site through AMPK and inhibit cell migration *in vitro*. With vascular smooth muscle lineage tracking mice, we found that VSMCs from media contribute to neointima formation after artery injury, and metformin reduces VSMC migration and the area of the neointima. Using streptozotocin (STZ)-induced diabetic ApoE^−/−^ mice, we found that metformin reduces atherosclerotic plaques, while S177A-Pdlim5, an unphosphorylatable mutant that carried adenovirus, undermines metformin's anti-atherosclerosis function. Taken together, metformin reduces the motility of vascular smooth muscle cells through the activation of the AMPK–Pdlim5 pathway, which contributes to the protective effects of metformin against diabetes-accelerated atherosclerosis and is beneficial for the therapy of metabolic syndrome.

## Materials and Methods

### Animal Experiments

Eight-week-old male ApoE^−/−^ mice (body weight, 20–25 g) on C57/BL6 background were purchased from Beijing Biocytogen Co., Ltd. (Beijing, China) and kept with free access to water and food in a specific pathogen-free room under 24°C and 12-h light/dark cycle at the laboratory animal center of Southern Medical University. ApoE^−/−^ mice were injected intraperitoneally with 50 mg/kg STZ for 5 days to induce DM. At 2 weeks later, the diabetic mice were randomly divided into eight groups (*n* = 10 per group): control group, metformin hydrochloride (*via* gastric gavage, 300 mg/kg/day, Sigma-Aldrich) group, wild-type Pdlim5 (Pdlim5 WT) that carried adenovirus (Ad) group, Ad Pdlim5 WT and Met group, Ad Pdlim5 S177A group, Ad Pdlim5 S177A and Met group, Ad Pdlim5 S177D group, and Ad Pdlim5 S177D and Met group. At 3 days after virus infection, the mice were fed with a high-fat diet subsequently. At the end of the experiments, the mice were euthanized with terminal anesthetic (isoflurane >4% in 95% O_2_ and 5% CO_2_). All the animal experiments were approved and performed according to the Institutional Animal Care and Use Committee (IACUC) of Southern Medical University, which conformed to the guidelines from Directive 2010/63/EU of the European Parliament on the protection of animals used for scientific purposes. The high-fat and high-cholesterol diets were purchased from Guangdong Experimental Animal Center. The 2-kg pack of high-fat diet (HFD) contains 4.4 kcal/g of energy, and the components per pack as listed by the manufacturer were as follows: 17% lard, 1.2% cholesterol, 0.2% sodium cholate, 10% casein, 0.6% calcium hydrogen carbonate, 0.4% stone powder, 0.4% premix, and 52.2% basic feed.

### Blood Glucose and Plasma Lipid Measurements

Blood glucose level was determined 2 weeks later after STZ induction with OneTouch Ultra2 Glucose Monitors (LifeScan, Milpitas, CA, USA). Mice whose blood glucose level was above 16.6 mmol/L were diagnosed as having DM. Plasma total cholesterol, triglyceride, low-density lipoprotein cholesterol, and high-density lipoprotein cholesterol were determined with biochemical kits (Jiancheng Biotechnology, Nanjing, China).

### Carotid Artery Injury

Twelve- to 15-week-old *Myh11-cre-EGFP* male mice, with background in C57BL/6 WT, were purchased from Shanghai Model Organisms Center, Inc. The carotid arterial intima of the mice were mechanically damaged with a beaded guidewire as described in the reference (25). The mice were anesthetized by inhalation with a mixture of isoflurane (2%) and oxygen (98%). The animal work was also approved and performed according to the IACUC of Southern Medical University, which conformed to the guidelines from Directive 2010/63/EU of the European Parliament on the protection of animals used for scientific purposes.

### Tissue Collection, *En face* Analysis of the Aortic Arch, and Immunohistochemical Staining

The mice were perfused with phosphate-buffered saline (PBS), followed by 4% paraformaldehyde, after euthanasia. The hearts, together with a short segment of the aorta, were collected and embedded or quickly frozen. Immunohistochemical staining was performed as described previously (26). All immunofluorescence micrographs and images of Oil Red O-stained areas of the atherosclerotic lesion were acquired with an Olympus FV1000 confocal laser scanning microscope (Olympus, Tokyo, Japan), and morphometric analysis was performed using ImageJ software (NIH).

### Western Blotting

Western blotting was performed according to the description in the reference (21).

### Cell Culture

The mouse aortic smooth muscle cells and 293T cells were purchased from the American Type Culture Collection. The cells were cultured in DMEM supplemented with 10% serum (Gibco) under a humidified environment at 37°C in 5% CO_2_ and 95% air. The cells were sub-cultured when grown to 80–90% confluence. Cells within 10 generations were used for the experiments. The AMPK-α1 knock-out knockdown-rescue (KDR)/EGFP-Pdlim5 VSMC was a gift generously provided by Professor Takashima (Osaka University).

### Adenoviral Infection

All adenoviruses, a replication-defective adenoviral vector expressing wild-type Pdlim5 or Pdlim5 mutant fused with flag or EGFP tag, were gifts from the Takashima group (Osaka University). The Pdlim5 S177A or S177D adenoviral vector expressed a mutant of Pdlim5 in which serine 177 was substituted with alanine (S77A) or aspartate (S77D), respectively. For the animal experiments, the diabetic mice were infected with adenovirus in an open-chest myocardium injection after having been anesthetized with a mixture of isoflurane (2%) and oxygen (98%). For the cellular model work, VSMCs were infected with Ad-Pdlim5 WT, Ad-Pdlim5-S177A, or Ad-Pdlim5-S177D overnight in a medium supplemented with 2% fetal calf serum. The cells were then washed and incubated in fresh VSMC growth medium without fetal calf serum for an additional 12 h prior to experimentation. These conditions typically produced an infection efficiency of at least 80% as determined by EGFP or flag expression.

### Scratch Assay

The mouse aortic smooth muscle cells were seeded on 35-mm glass dishes at an initial density of 5 × 105 cm^2^. A scratch was made with a P-200 pipette tip 8 h after seeding. Then, 1 mM of metformin hydrochloride (Sigma-Aldrich) was added after changing the medium, with PBS as the control. The lesions were observed with a Zeiss inverted microscope, measured with the ImageJ software (NIH) once per hour, and totally observed for 8 h.

### Time-Lapse Imaging of VSMC Cells

EGFP-Pdlim5-WT cells or EGFP-Pdlim5/AMPK-α1 KO cells were plated on 35-mm glass dishes coated with collagen at an initial density of 4 × 104 cm^2^. At 5 h after plating, the cells were treated with metformin (1 mM). The fluorescence images were recorded as described before (21).

### Statistical Analyses

The data in the graphs are presented as means ± SEM. Two-tailed Student's *t*-test was utilized to compare the two groups. Differences among multiple experimental groups were analyzed by one-way analysis of variance, followed by a *post-hoc* comparison with Dunnett's method utilizing SPSS 16 (IBM). *P* < 0.05 was considered statistically significant (^*^P < 0.05, ^**^P < 0.01; ^***^P < 0.001).

## Results

### Metformin Inhibits VSMC Migration Through the AMPK–Pdlim5 Pathway and Independent of VSMC Phenotype Transition

**Figure 1:**
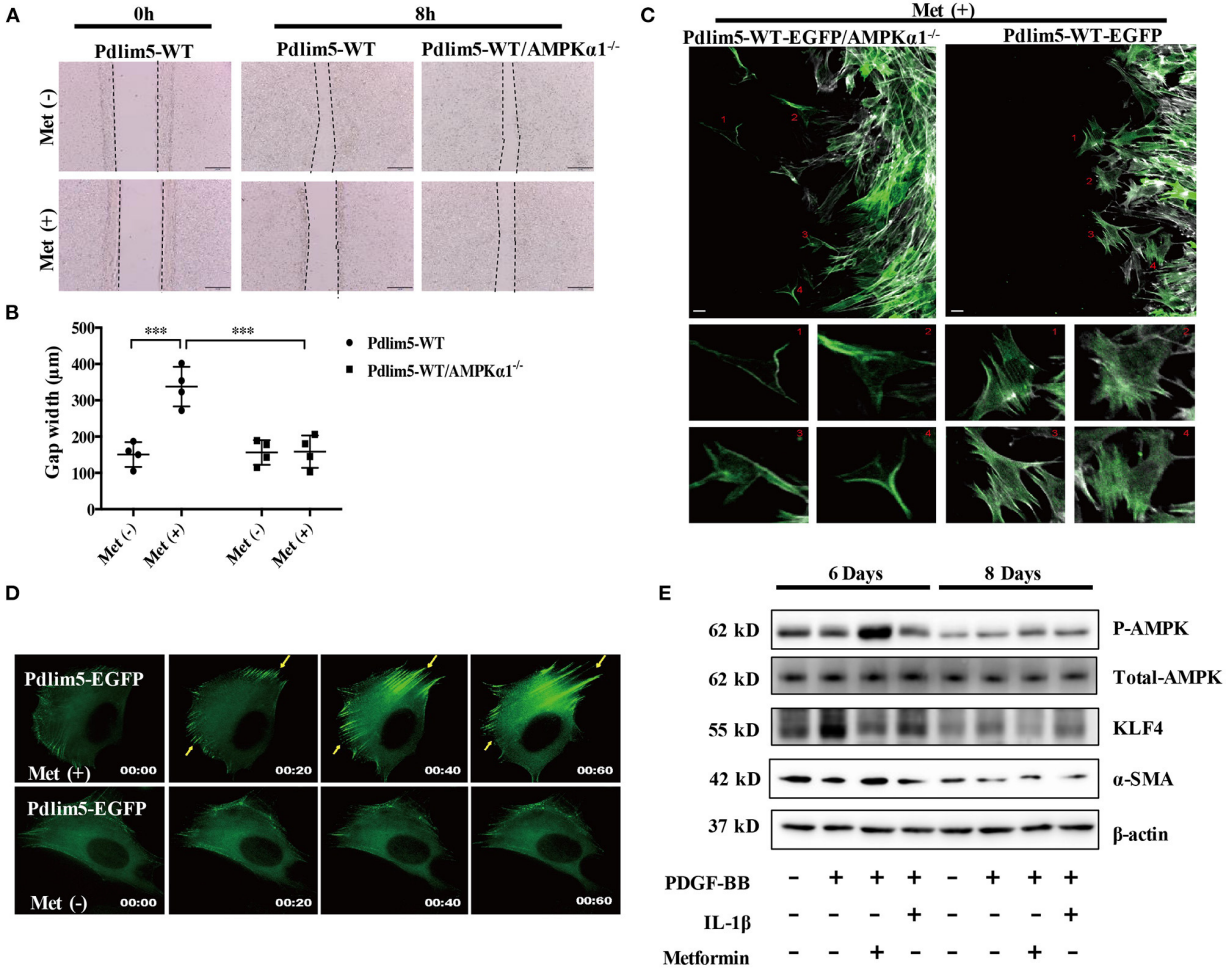
Metformin inhibits vascular smooth muscle cell (VSMC) migration through the AMPK–Pdlim5 pathway. **(A)** EGFP-Pdlim5 VSMCs and AMPK *α*1-null/EGFP -Pdlim5 VSMCs were stimulated with metformin (1 mM) for 8 h in a scratch assay. **(B)** Bar graph showing the gap width 8 h after scratching [from **(A)**]. Data are representative of means ± SEM from four independent experiments. The significance of differences between series of results was assessed using one-way analysis of variance, followed by a *post-hoc* comparison with Dunnett's method for multiple comparisons. ^***^P < 0.001, *n* = 4. **(C)** GFP images of AMPK *α*1-null/KDR/EGFP-WT-Pdlim5 cells before and after metformin stimulation (1 mM). The magnified images below show the cells labeled by the number. Scale bar, 10 mm. **(D)** Time-lapse images of GFP signal in EGFP-WT-Pdlim5 cells with or without metformin stimulation (1 mM). Yellow arrowheads indicate the growth of dorsal stress fibers from the opposite side in EGFP-WT-Pdlim5 cells after metformin stimulation. Scale bar, 10 mm. **(E)** Western blot of vascular smooth muscle cell (VSMC) contractile markers SMA, transcriptional regulator KLF4, and phosphorylated AMPK in control, PDGF-BB-treated, metformin combined with PDGF-BB and IL-1β combined with PDGF-BB-treated VSMCs.

**Figure 2:**
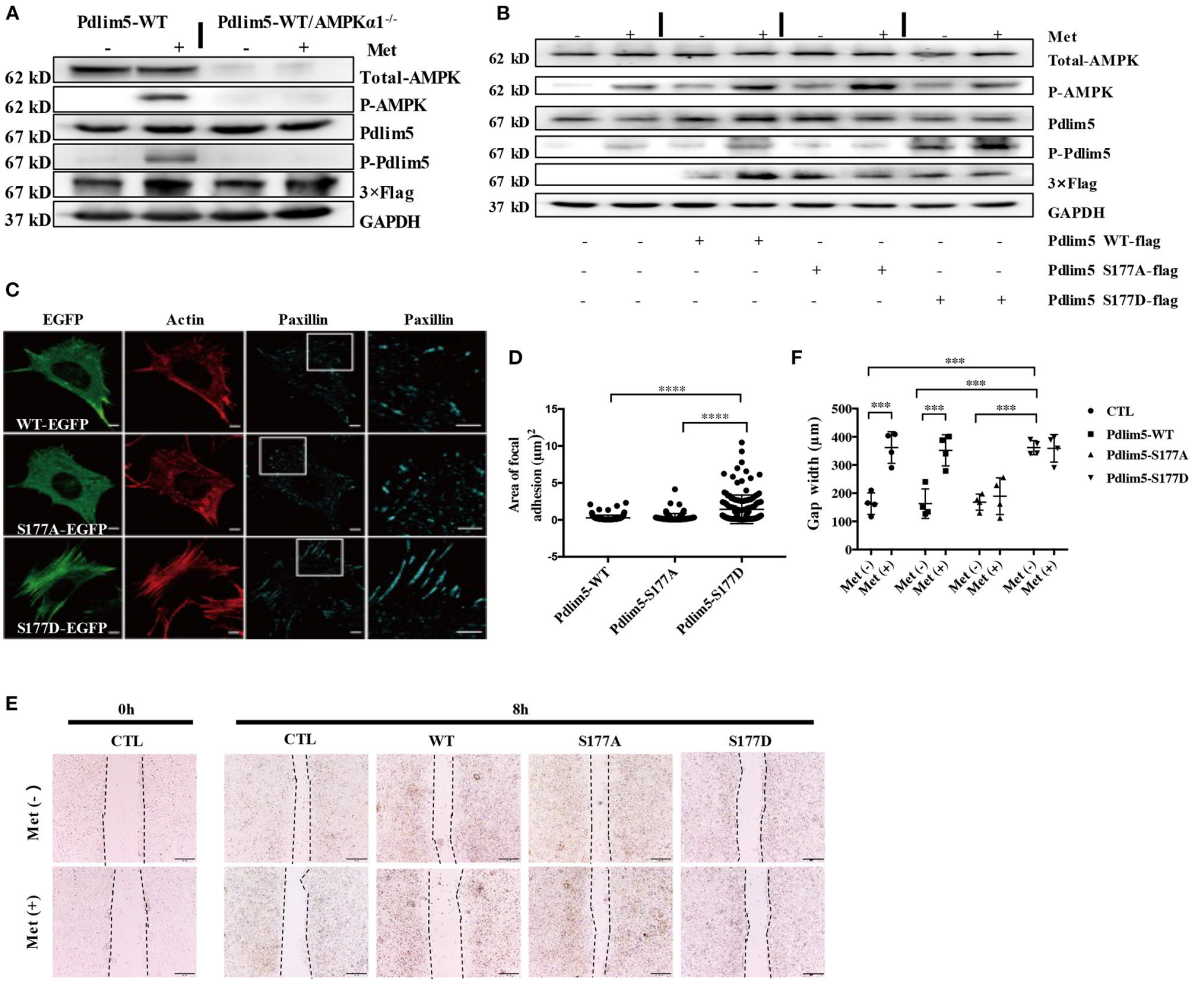
Metformin inhibits lamellipodia formation and enforces focal adhesions in vascular smooth muscle cells (VSMCs) through the AMPK–Pdlim5 pathway. **(A)** Flag-WT-Pdlim5 VSMCs and AMPK *α*1-null/EGFP-WT-Pdlim5 VSMCs were stimulated with metformin before detecting the phosphorylation level of AMPK and Pdlim5 with immunoblotting. **(B)** Metformin activates the AMPK–Pdlim5 signaling pathway. VSMCs were transfected with CTL siRNA or Pdlim5 siRNA. Pdlim5 siRNA-resistant flag-Pdlim5 (WT, S177A, or S177D) was added back by adenoviral-mediated gene delivery. The phosphorylation levels of AMPK and Pdlim5 were detected with western blotting. **(C)** GFP signal and immunostaining of actin and paxillin in KDR/EGFP-Pdlim5 cells (WT, S177A, and S177D). Actin and paxillin were stained with phalloidin and anti-paxillin antibody to display actin microfilaments and focal adhesions, respectively. The right panels are the magnified images of the region outlined by white boxes. Scale bar, 10 mm. **(D)** The focal adhesion area of EGFP-Pdlim5 cells (WT, S177A, and S177D) were measured according to the paxillin-positive region in **(B)** and compared using one-way analysis of variance, followed by a *post-hoc* comparison with Dunnett's method for multiple comparisons. ^****^P < 0.0001, *n* = 100. **(E)** Scratch assay of metformin-stimulated EGFP-Pdlim5 cells. Phase-contrast images of EGFP-Pdlim5 cells (WT, S177A, and S177D) 8 h after scratching with or without metformin. Scale bar, 0.5 mm. **(F)** Bar graph showing the gap width 8 h after scratching [from **(E)**]. Data are representative of means ± SEM from four independent experiments. The significance of the results was assessed using one-way analysis of variance, followed by a *post-hoc* comparison with Dunnett's method for multiple comparisons. ^***^P < 0.001, *n* = 4.

To investigate the role of the AMPK–Pdlim5 pathway in cell migration, we established the KDR system in wild-type VSMCs or AMPKα1 null VSMCs as described before (21), in which endogenous Pdlim5 was replaced with EGFP or flag-fused Pdlim5, Pdlim5 S177A (an unphosphorylatable mutant), or Pdlim5-S177D (a phosphomimetic mutant), respectively (Figures 1, 2).

In the scratch assay, it was observed that metformin inhibits the wounding healing ability of WT-Pdlim5 VSMCs. However, metformin failed to inhibit wound healing in the AMPKα1 absent WT-Pdlim5 VSMCs (Figures 1A,B). Enhanced stress fiber and reduced VSMC migration were also observed with time-lapse imaging (Figure 1C). It was shown that metformin reduced lamellipodia formation and promoted the enhancement of the EGFP signals from the side opposite to the lamellae, a pattern similar to the growth of dorsal stress fibers (Figure 1C).

It has been reported that the migration of VSMCs is related to phenotype transition, which means the loss of contractile proteins and the expression of specific transcriptional factors. To investigate whether metformin inhibit VSMC migration by influencing phenotype switching, wild-type VSMCs were treated with PDGF-BB, a phenotype transition inducer, or PDGF-BB combined with metformin or inflammatory cytokine IL-1 as a control for 8 days. It was shown that PDGF-BB increased the expression of phenotype switching regulator KLF4 from day 6, reducing the expression of contractile protein SMA from day 8. However, metformin activated AMPK effectively and reduced the KLF4 expression but had no significant influence upon SMA expression (Figure 1E). These results suggest that metformin inhibits VSMC migration through AMPK activation but not via the phenotype transition pathway.

### Metformin Inhibits VSMC Migration Through the Enforcement of Focal Adhesions and Reducing Lamellipodia Formation

To investigate whether metformin could induce the phosphorylation of Pdlim5 through AMPK, EGFP or flag-fused WT-Pdlim5 Pdlim5, Pdlim5 S177A (an unphosphorylatable mutant), or Pdlim5-S177D (a phosphomimetic mutant) were overexpressed in wild-type VSMCs or AMPKα1 null VSMCs before metformin exposure. In Figure 2A, it was shown that metformin could induce the phosphorylation of AMPK and Pdlim5 in wild-type VSMCs but not in WT-Pdlim5/AMPKα1 null VSMCs. The phosphorylation of Pdlim5 was not observed in Pdlim5-S177A VSMCs, while Pdlim5-S177D was recognized by the Ab-pS177 antibody even without Met stimulation (Figure 2B). In Figure 2C, both WT- and Pdlim5-S177A cells possessed smooth lamellipodia-like edges, whereas Pdlim5-S177D cells displayed decreased lamellipodia formation and jagged edges with excessive filopodia-like protrusions and ventral stress fibers. In addition, it was found that both WT- and S177A-Pdlim5 cells had tiny and scattered spots of focal adhesions at the junction between the lamellipodia and lamella; by contrast, in S177D-Pdlim5 cells, focal adhesions were displaced to the edge of the cell and significantly enlarged in size (Figure 2C).

Next, a scratch assay was performed to observe whether decreased lamellipodia and enhanced stress fiber would inhibit cell migration (Figure 2E). It was shown that the wound healing ability of S177D cells is lower than that of WT- and Pdlim5-S177A cells, while Met inhibited the migration of both WT- and Pdlim5-S177D cells only, except S177A cells (Figure 2E).

### Metformin Attenuates Intimal Hyperplasia After Artery Injury in Myh11-Cre/Rosa26-EGFP Mice

**Figure 3:**
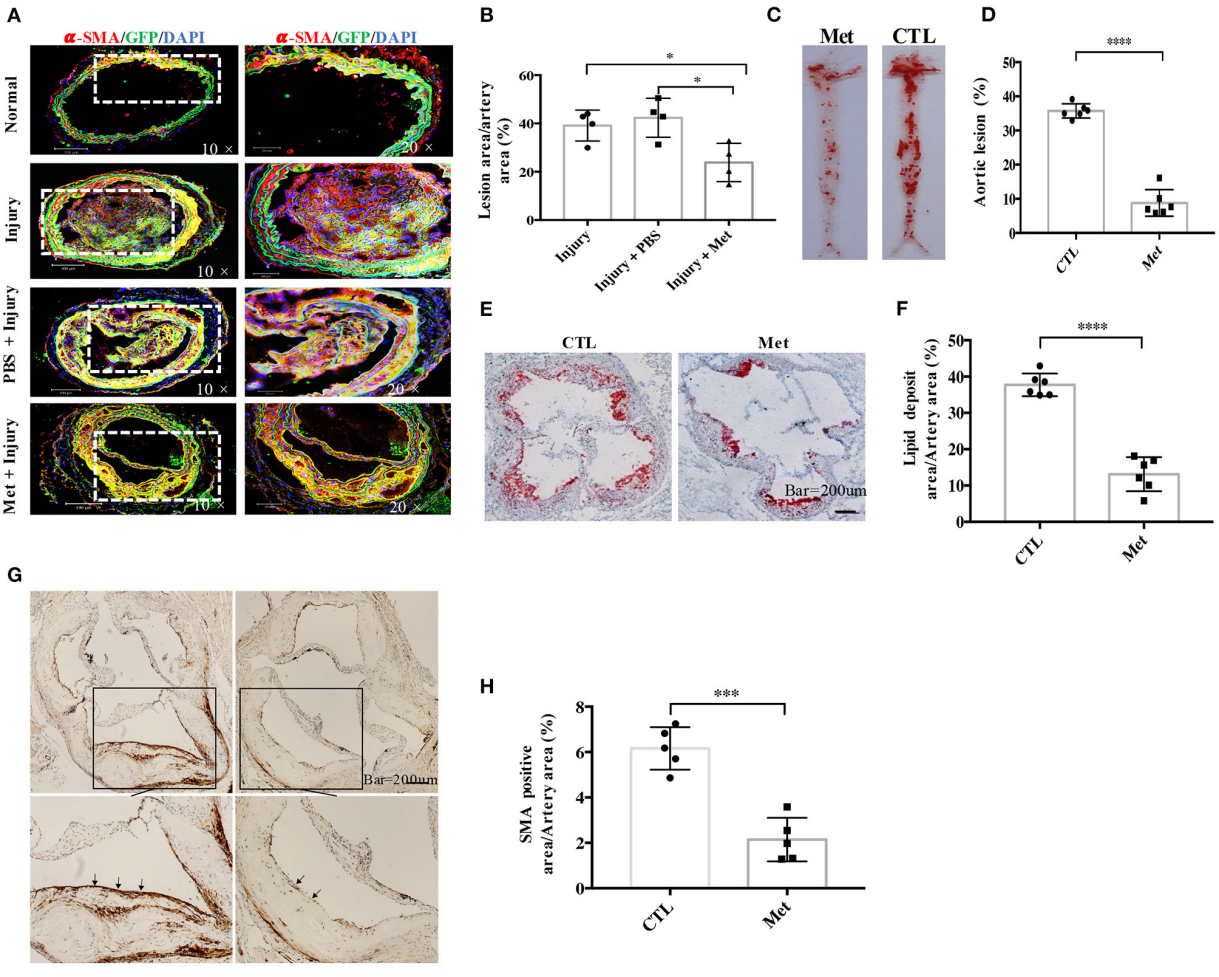
Metformin inhibits medial vascular smooth muscle cell (VSMC) migration *in vivo* and reduces VSMC infiltration in atherosclerotic plaques in ApoE^−/−^ mice. **(A)** Myh11cre-eGFP mice were treated with saline or metformin for a week after left carotid wire injury (*n* = 5 per group). Representative images of the immunostaining of SMA- and EGFP-positive cells in an artery cross-section. **(B)** Quantitative analysis of the percentage of lesion area compared to the cross-sectional area of the artery. Data are representative of means ± SEM from five independent experiments. The significance of differences between the series of results was assessed using one-way analysis of variance, followed by a *post-hoc* comparison with Dunnett's method for multiple comparisons. ^*^P < 0.05, *n* = 5. **(C)** ApoE^−/−^ mice were injected with streptozotocin for 5 days to induce diabetes and then fed with high-fat diet and metformin intervention (Met) or control (CTL) for 8 weeks. The atherosclerotic lesions in the aorta were stained with Oil Red O. **(D)** Quantification of the *en face* lesion area in the aorta. The significance of differences was assessed using one-way analysis of variance, followed by a *post-hoc* comparison with Dunnett's method for multiple comparisons. ^****^P < 0.0001, *n* = 6. **(E)** Oil Red O staining of atherosclerotic lesions at the aortic root. **(F)** Quantification of the lesion size in the aortic root. The significance of differences was analyzed using one-way analysis of variance, followed by a *post-hoc* comparison with Dunnett's method for multiple comparisons. ^****^P < 0.0001, *n* = 6. **(G)** Immunochemistry staining of α-SMA in the aortic root. **(H)** Quantitative analysis of α-SMA-positive area in the aortic root. The significance of differences between series of results was assessed using one-way analysis of variance, followed by a *post-hoc* comparison with Dunnett's method for multiple comparisons. ^***^P < 0.001, *n* = 5.

As metformin could inhibit VSMC migration *in vitro*, a wire injury-induced vascular remodeling model was utilized to verify this function of Met *in vivo* (Figure 3). To accomplish this experiment, 6- to 8-week-old Myh11-Cre/Rosa26-EGFP mice, which express EGFP in mature VSMCs, were subjected to ligation of the left carotid artery. An accumulation of VSMC-derived cells in the intimal hyperplasia of the left carotid artery was observed by confocal microscopy with arterial cross-sections. It was found that many Myh11-expressing VSMC-derived EGFP-positive cells contributed to the neo-intima lesions and constituted a significant proportion of the total cell number within lesions. Interestingly, metformin treatment reduced the intima hyperplasia (Figure 3A) and patch size (Figure 3B) than the saline treatment significantly.

### Metformin Alleviates Atherosclerotic Lesions in Diabetic ApoE^–/–^ Mice

To study whether metformin could be used to prevent diabetes-accelerated atherosclerosis, DM and atherosclerosis were induced in ApoE^−/−^ mice with streptozocin and HFD. The diabetic mice were treated with metformin as described in “Materials and Methods.” It was found that the diabetic ApoE^−/−^ mice possessed obvious *en face* lesions in the aortic arch and thoracic and abdominal aorta and greater atherosclerotic lesions in the aortic root (Figures 3C–E). Metformin intervention significantly reduced the lesion areas in the aortic root and the aortic arch in diabetic ApoE^−/−^ mice. The SMA-positive deposition in the aortic root was also smaller in metformin-treated mice than in diabetic mice, which suggests that metformin inhibited VSMC accumulation in atherosclerotic plaques (Figures 3F,G).

### The Activation of the AMPK–Pdlim5 Pathway Is Involved in the Protective Function of Metformin Against Diabetes-Accelerated Atherosclerosis in ApoE^–/–^ Mice

**Figure 4:**
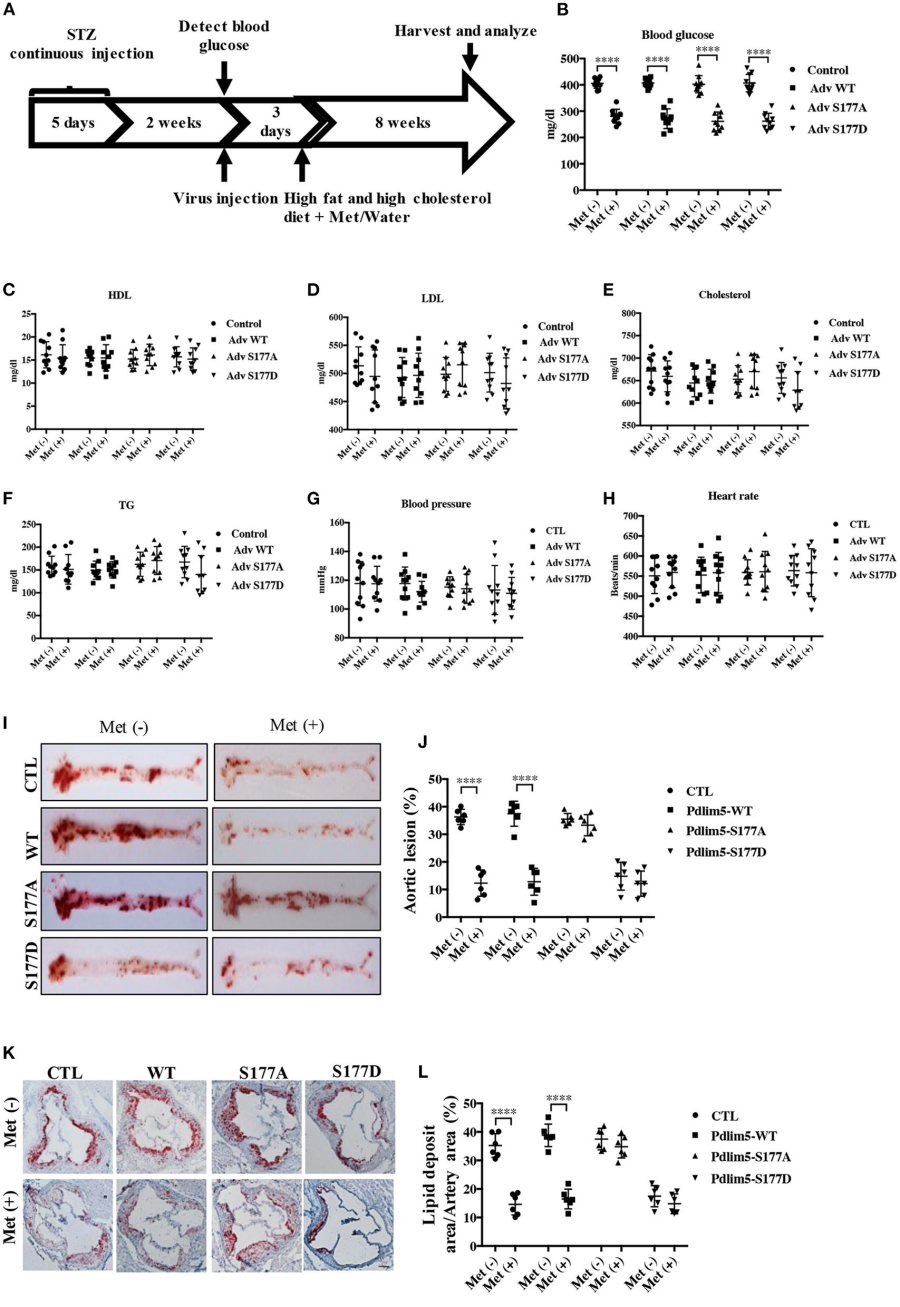
Phosphorylation of Pdlim5 is involved in the anti-atherosclerosis function of metformin in diabetic ApoE^−/−^ mice. The ApoE^−/−^ mice had induced diabetic atherosclerosis and intervened with metformin as described in “Materials and Methods” for 8 weeks (*n* = 10 per group). At the same time, the mice were randomly separated into four groups: vehicle group, adenovirus WT Pdlim5 group, Ad S177A Pdlim5 group, and Ad S177D Pdlim5 group. **(A**–**H)** Blood glucose, plasma HDL, LDL, cholesterol, triglyceride, blood pressure, and heart rate were determined with a commercial kit. The data are representative of means ± SEM from 10 independent experiments. The significance of differences between the series of results was assessed using one-way analysis of variance, followed by a *post-hoc* comparison with Dunnett's method for multiple comparisons. ^****^P < 0.0001, *n* = 10. **(I)** Oil Red O staining of atherosclerotic lesions at the aorta. **(J)** Quantification of the *en face* atherosclerotic lesion area in the aorta. Data are representative of means ± SEM from six independent experiments. The significance of differences was assessed using one-way analysis of variance, followed by a *post-hoc* comparison with Dunnett's method for multiple comparisons. ^****^P < 0.0001, *n* = 6. **(K)** Oil Red O staining of atherosclerotic lesions at the aortic root. **(L)** Quantification of the atherosclerotic lesion size in the aortic root. Data are representative of means ± SEM from six independent experiments. The significance of differences of the results was assessed using one-way analysis of variance, followed by a *post-hoc* comparison with Dunnett's method for multiple comparisons. ^****^P < 0.0001, *n* = 6.

**Figure 5:**
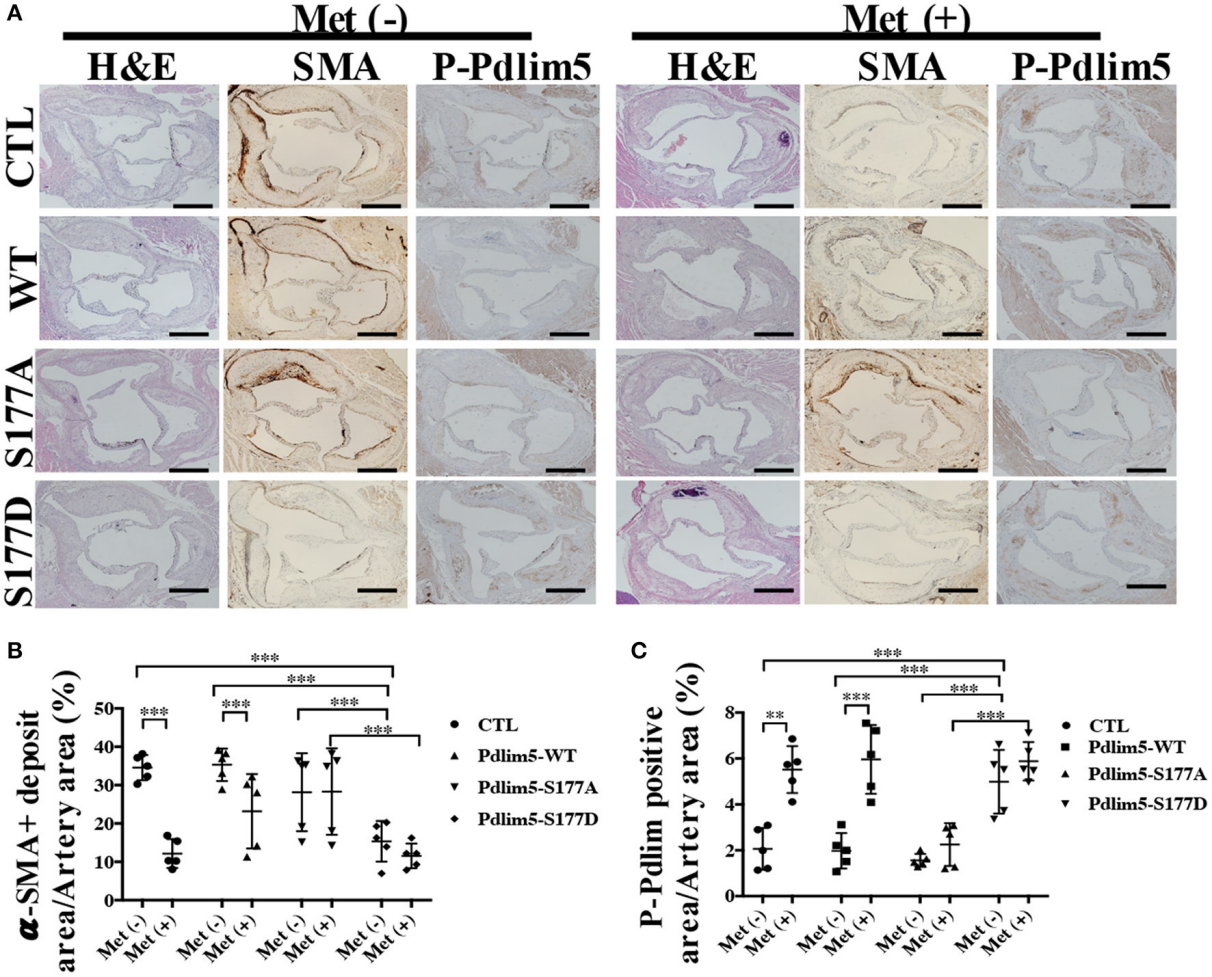
The phosphorylation of Pdlim5 induced by AMP-activated protein kinase was related to metformin's anti-atherosclerosis function. **(A)** Representative images of H&E staining and immunostaining of α-SMA and phosphorylated Pdlim5 at the aortic root of diabetic ApoE^−/−^ mice. Scale bar = 200 μm. **(B)** Quantification of α-SMA-positive area in the aortic root. Data are representative of means ± SEM from five independent experiments. The significance of differences between the series of results was assessed using one-way analysis of variance, followed by a *post-hoc* comparison with Dunnett's method for multiple comparisons. ^***^P < 0.001, *n* = 5. **(C)** Quantification of phosphorylated Pdlim5-positive area in the aortic root. Data are representative of means ± SEM from five independent experiments. The significance of differences was assessed using one-way analysis of variance, followed by a *post-hoc* comparison with Dunnett's method for multiple comparisons. ^**^P < 0.01, ^***^P < 0.001, *n* = 5.

To study the role of AMPK–Pdlim5 pathway in the development of atherosclerosis, ApoE^−/−^ animals were divided randomly into streptozocin-induced diabetes mellitus together with or without metformin, Pdlim5 phosphomimetic mutant that carried adenovirus (Pdlim5 S177D) or Pdlim5 unphosphorylatable mutant that carried adenovirus (Pdlim5 S177A). It is shown in Figure 4 that metformin reduced hyperglycemia significantly (Figure 4B), while it did not exhibit an obvious influence upon dyslipidemia induced by STZ and HFD (Figures 4C–F). However, the manipulation of Pdlim5 phosphorylation with adenovirus has no significant influence on metabolic disorders in DM mice (Figures 4B–F). The diabetic ApoE^−/−^ mice developed significantly larger *en face* lesions in the aortic arches (Figures 4G–J) and larger SMA- and phosphorylated Pdlim5-positive lesions in the aortic roots compared with those in the metformin treatment group (Figures 5A–C). The Pdlim5-negative mutant adenovirus (S177A) alleviated the protective function of metformin as mentioned above and increased the plaque area significantly (Figures 4G–J, 5A–C). However, Pdlim5 constitutively active adenovirus (S177D) suppressed atherosclerosis obviously even under the absence of metformin (Figures 4G–J, 5A–C).

## Discussion

The blood vessels mainly contain two cell types: endothelial cells (EC) and vascular smooth muscle cells. Injury of the endothelium leads to the initiation of atherosclerosis, while the abnormal proliferation and migration of vascular smooth muscle cells result to the development of atherosclerotic plaques (27). An aberrant EC–VSMC interaction could promote atherogenesis (27). It is widely accepted that the effects of endothelial dysfunction on VSMCs are reduction of NO bioavailability and/or augmentation of vasoactive constrictors released from the endothelium (28). The injured EC can also recruit inflammatory cells and release cytokines that induce a phenotype change of VSMCs from the “contractile” phenotype to the “synthetic” state that can migrate and proliferate from the media to the intima (29). An accumulation of VSMC in the vascular intima is a hallmark of atherosclerosis, but their exact origins are still in controversy (8, 22). In humans, both pre-existing intimal and medial VSMCs can contribute to plaque VSMCs (30). In mice, the VSMCs in the fibrous cap are unambiguously derived from media, which suggests the importance of cell migration in the pathogenesis of atherosclerosis (31). This is consistent with our finding that inhibiting VSMC migration through the activation of AMPK reduces neointima formation induced by artery injury and STZ/HFD-induced atherosclerosis. The mechanics of VSMC migration in atherosclerotic lesions involves the formation of plasma membrane-leading lamellae (leading edge) and the disengagement of focal adhesions that are in contact with the ECM (32). We found that metformin could inhibit cell migration through the enforcement of focal adhesions and reducing lamellipodia formation *in vitro*. The underlying mechanism has been elucidated by our publication and those of others, such that phosphorylation of Pdlim5 by AMPK disrupts the binding between Pdlim5 with Arhgef6 at the cell periphery (21). The dissociation suppresses Rac1 activity and dislocates the Arp2/3 complex from the leading edge of cells which impairs lamellipodia formation and cell migration (21, 32, 33).

VSMC migration in atherosclerosis has also been related to phenotype switching—a synthetic, de-differentiated state (34). The phenotype-switched VSMCs exhibit a reduced production of contractile proteins but with a higher expression of ECM-related products and increased levels of secretory organelles and pro-inflammatory cytokines (35). At the molecular level, the VSMC phenotype transition is governed by transcription factors myocardin serum response factor 58 and Krüppel-like factor 4 (KLF4) (23, 36). Diabetes mellitus-associated pathological factors exacerbate the synthetic phenotype of VSMCs through the up-regulation of KLF4 (37). Although our work has shown that metformin-induced AMPK activation has an insignificant influence on the expression of KLF4 and “contractile proteins” (Figure 2E), the relationship between VSMC migration and phenotype switching in atherosclerotic plaques still needs further investigation.

AMPK is a vital enzyme for regulating cellular energy homeostasis (38). The activation of AMPK depends on phosphorylation at its T172 site and binding with AMP and/or ADP (39). Compelling evidence has indicated an inverse correlation between diabetes and AMPK activity (38). Therefore, AMPK-activating agents have the potential to be utilized as precaution or therapies against diabetes and DM-related complications. Indeed metformin, an indirect AMPK activator and well-known T2DM drug, could reduce atherosclerosis in patients with diabetes (19, 39). However, the underlying mechanism is not clear yet. Recent work found that AMPK also plays an important role in the regulation of cell polarity and motility (21), which throws a light on the research of metformin's anti-atherosclerosis function. There are two different α isoforms (α1 and α2) that are differentially expressed in different tissues. Several references found that AMPKα2 plays an important role in the aberrant migration of vascular smooth muscle cells in atherosclerosis. However, our previous work found that AMPKα1 is more important for the migration of vascular smooth muscle cells *in vitro* (21), and the phosphorylation of Pdlim5 by AMPKα1, but not AMPKα2, plays an important role in the anchoring of vascular smooth muscle cells (21). In this study, we found that metformin activates AMPK, which phosphorylates Pdlim5 at Ser177, resulting in the attenuation of lamellipodia formation and the inhibition of vascular smooth muscle cell migration from the medial to intima. We also demonstrated that metformin reduces VSMC accumulation in atherosclerotic plaques *via* an AMPK–Pdlim5-dependent manner in STZ- and HFD-induced diabetic ApoE^−/−^ mice. It is consistent with the existing concept that metformin has multiple beneficial effects on vascular cells (endothelial cells, vascular smooth muscle cells, and macrophages), many of which are AMPK-mediated (15, 40–43).

Our previous work found that AMPK has many substrates—CLIP170, VASP, and Pdlim5, for instance (21, 44, 45), but their inductions need a different activated level of AMPK. Basically, AMPK has three activated levels which are low activity, physiological activity, and augmented activity (21). Different activated AMPK situations induce different substrates, but only physiologically activated AMPK stimulates cell migration, while others both inhibit migration (21). Less activated AMPK inhibits migration through CLIP170, which is a component of a microtube (21), while augmented activated AMPK inhibits cell migration through the phosphorylation of Pdlim5, which is a component of actin (21). Metformin, as a well-accepted oral drug for type 2 diabetes patients, could induce the augmented activation of AMPK *in vitro* and *in vivo* efficiently (46), so our work first found that Pdlim5, a component of muscle cytoskeleton that is related to many cardiovascular diseases (47), as a substrate of AMPK could be phosphorylated only by augmented activated AMPK, which induces the abolishment of the migratory machine in VSMCs *in vitro* and *in vivo*. This work partially explains the beneficial effects of metformin toward diabetes-accelerated atherosclerosis. This work suggests that Pdlim5 has the potential to be a drug target to suppress the development of atherosclerosis. Moreover, we also found that Pdlim5 plays an important role in the pathology of some cardiovascular diseases through other post-translational modifications instead of phosphorylation. Hence, there are still lots of work to be accomplished to translate the knowledge of atherogenesis in the clinic.

## Conclusion

Nevertheless, our research revealed that augmentation of AMPK activity could inhibit VSMC migration from the media to the atherosclerotic plaques through the phosphorylation of Pdlim5. It may be useful to develop novel therapies toward atherosclerosis and other complications in diabetes.

## Data Availability Statement

The original contributions presented in the study are included in the article/supplementary material, further inquiries can be directed to the corresponding author/s.

## Ethics Statement

The animal study was reviewed and approved by Institutional Animal Care and Use Committee (IACUC) of Southern Medical University.

## Author Contributions

YY, WW, and ZZ conceived and designed the studies, wrote the manuscript, and completed all analyses. YY and TL performed the experiments and data analysis. ZL, MH, DW, YX, XY, YB, and YL contributed to the acquisition of data. All authors contributed to the article and approved the submitted version.

## Conflict of Interest

The authors declare that the research was conducted in the absence of any commercial or financial relationships that could be construed as a potential conflict of interest.

## Publisher's Note

All claims expressed in this article are solely those of the authors and do not necessarily represent those of their affiliated organizations, or those of the publisher, the editors and the reviewers. Any product that may be evaluated in this article, or claim that may be made by its manufacturer, is not guaranteed or endorsed by the publisher.

## Abbreviations

AMPK: AMP-activated protein kinase
CTL: control
CVD: cardiovascular disease
DM: diabetic mellitus
ECM: extracellular matrix
KDR: knockdown-rescue
LDL: low-density lipoprotein
STZ: streptozotocin
Met: metformin
Pdlim5: PDZ and LIM domain 5
T2DM: type 2 diabetic mellitus
VSMCs: vascular smooth muscle cells.
